# Microglial Activation in Cerebrovascular Accidents and the Manifestation of Major Depressive Disorder: A Comprehensive Review

**DOI:** 10.3390/brainsci16010063

**Published:** 2025-12-31

**Authors:** Karla Cristina Razón-Hernández, Gabriela Martínez-Ramírez, Javier Villafranco, Oscar Rodríguez-Barreto, Daniel Ortuño-Sahagun, Roxana Magaña-Maldonado, Karla Sánchez-Huerta, Enrique Becerril-Villanueva, Lenin Pavón, Enrique Estudillo, Gilberto Pérez-Sánchez

**Affiliations:** 1 Laboratorio de Neurociencias, Subdirección de Medicina Experimental, Instituto Nacional de Pediatría, Mexico City 04530, Mexico; neuroenlace@hotmail.com (K.C.R.-H.); ksanchezh@pediatria.gob.mx (K.S.-H.); 2 Laboratorio de Psicoinmunología, Instituto Nacional de Psiquiatría Ramón de la Fuente Muñiz, Mexico City 14370, Mexico; gaby9497ram@gmail.com (G.M.-R.); villafrancoj20@gmail.com (J.V.); osckaros@gmail.com (O.R.-B.); lusenbeve@inprf.gob.mx (E.B.-V.); lkuriaki@inprf.gob.mx (L.P.); 3 Laboratorio de Reprogramación Celular, Instituto Nacional de Neurología y Neurocirugía Manuel Velasco Suárez, Mexico City 14269, Mexico; rmagana@innn.edu.mx (R.M.-M.); 4 Facultad de Estudios Superiores Zaragoza, Universidad Nacional Autónoma de México, Mexico City 04510, Mexico; 5 Laboratorio de Neuroinmunobiología Molecular, Instituto de Neurociencias Translacionales, Centro Universitario de Ciencias de la Salud, Universidad de Guadalajara, Guadalajara 44100, Mexico; daniel.ortuno@academicos.udg.mx (D.O.-S.)

**Keywords:** microglial activation, major depressive disorder, cerebrovascular accidents, vascular depression, MyD88

## Abstract

Emerging evidence highlights a strong association between cerebrovascular accident (CVA) and major depressive disorder (MDD), mediated by immune dysregulation. Elevated levels of proinflammatory cytokines, reduced adaptive immune responses, altered immune cell composition, and increased microglial activation characterize this bidirectional relationship. Microglial activation appears to be a central molecular mechanism linking CVA and MDD, underscoring the immune system’s crucial role in disease pathogenesis. This interplay suggests that immune-driven processes not only exacerbate neurological damage but also contribute to psychiatric manifestations. Based on current literature, the role of proinflammatory processes, particularly microglial activation, in the relationship between CVA and MDD warrants special attention. In this context, the participation of myeloid differentiation factor 88 (MyD88), a cytosolic adaptor protein, appears to play a key role in proinflammatory signaling pathways driving microglial activation. Thus, focusing on MyD88 emerges as a promising complementary strategy for future research and for advancing our understanding of the mechanisms underlying microglial homeostasis dysregulation and its link to the pathophysiology of MDD and CVA.

## 1. Introduction

Cerebrovascular accident (CVA) is defined as an acute episode of focal neurological dysfunction that persists for more than 24 h and it is divided into ischemic and hemorrhagic, according to the blood flow to the brain. Ischemic stroke is caused by deficient blood and oxygen supply to the brain and hemorrhagic stroke is caused by bleeding [1]. On the other hand, major depressive disorder (MDD) is a psychiatric disorder characterized by persistent disturbances of mood and cognitive functioning, accompanied by affective symptoms that are persistent. MDD and CVA are two conditions that represent a global public health problem [2,3,4]. In addition, it has been shown that cerebrovascular burden in midlife (≥65 age) predicts depressive symptomatology later in life, suggesting a relationship between the two conditions [5]. According to this relationship, the hypothesis of vascular depression has been suggested, which may be associated with CVA and the microglial activation. Therefore, this work compiles the studies of CVA, MDD, and their relationship with the hypothesis of vascular depression, as well as the role of the microglial proinflammatory activation in this pathological process.

## 2. Materials and Methods

In this comprehensive review we used PubMed, Scientific Electronic Library Online (SciELO), Scopus and Google Scholar databases, without restrictions on publication dates, including scientific articles in English and Spanish. The following keywords were used in the section CVA and MDD: Major Depression Disorder and Cerebral Vascular Accident. In the section Vascular Depression: vascular, depression, risk factors, cerebral small vessel disease and inflammatory responses. In the section microglial activation after CVA and MDD: microglial activation, blood-brain-barrier, receptors Toll-like, molecular mechanisms and neuroinflammatory response. We reviewed titles and abstracts of all retrieved articles and retained those that addressed CVA, MDD, or the relationship between these conditions; discussed neuroinflammatory mechanisms, microglial activation, immune dysregulation, or vascular depression in the context of stroke and/or depression; or provided original research, review articles, or mechanistic evidence relevant to our synthesis objectives. The potential publication bias associated with the exclusive use of indexed databases and the omission of gray literature represents a limitation of the study; however, our multi-database strategy and the inclusion of SciELO help to partially offset this limitation.

## 3. Cerebrovascular Accident and Major Depressive Disorder

### 3.1. Cerebrovascular Accident

The American Heart Association and American Stroke Association (AHA/ASA) define stroke as an acute episode of focal neurological dysfunction that persists for more than 24 h [6]. CVA is classified into two main types: ischemic and hemorrhagic. In both cases, the metabolic demands of the Central Nervous System (CNS) cannot be met, resulting in tissue damage and cell death. An Ischemic stroke is caused by an interruption of cerebral blood flow and oxygen supply to the brain, which is caused by thrombosis or embolism. A hemorrhagic stroke is caused by either a hemorrhage or an abnormally high permeability of the blood vessels [1,7]. Epidemiological data indicate that stroke is the second leading cause of mortality worldwide, responsible for the deaths of 6.6 million people. Additionally, it is the third leading cause of disability, accounting for 143 million disability-adjusted life years (DALYs). Over the past three decades, there has been a 70% increase in the overall incidence of stroke, an 85% increase in prevalence, and a 43% increase in mortality [8,9,10]. The most significant risk factors for the onset of stroke are elevated systolic blood pressure, elevated body mass index, elevated fasting plasma glucose concentrations, environmental pollution by particulate matter, and smoking [10,11,12]. These individuals often carry comorbidities such as hypertension, diabetes, or heart disease, which increase the risk of medical complications during recovery from a stroke [13].

### 3.2. Major Depressive Disorder (MDD)

MDD is a psychiatric disorder characterized by persistent disturbances in mood and cognitive functioning. Clinically, it presents with anhedonia (loss of interest or pleasure in previously rewarding activities) accompanied by affective and cognitive symptoms that persist for at least two weeks [2,3,4]. Epidemiological data from the World Health Organization (WHO) indicates that approximately 3.8% of the global population experiences depression. This equates to an estimated 280 million individuals worldwide who are affected by this mental health condition [14]. MDD is a common comorbidity in chronic diseases, affecting 2 to 3 times more women than men. The prevalence of MDD in some diseases ranges from: (1) 12 to 18% in diabetes (2) 15 to 23% in coronary heart disease, (3) 15 to 25%, in cancer (4) 20% to 50% in chronic kidney disease, and (5) 25% to 60% in chronic pain [15]. Also, depression is associated with a 1.43-times increased risk of all-cause mortality (HR 1.43; 95% CI, 1.27–1.60) and 1.13-times increased risk of developing stroke (HR 1.13; 95% CI, 1.00–1.28) [16]. The etiology of depression is multifactorial (social, psychological and biological). Some risk factors that influence the development of depression are diseases such as diabetes, hypertension, coronary artery disease, CVA, and pulmonary diseases, among others [17,18]. In addition, patients with MDD report elevated levels of proinflammatory cytokines [17,19], which modifies the innate and adaptive immune system response. All these together can exacerbate the symptoms of depression and the physiological response [20].

## 4. Vascular Depression

The evidence indicates that cerebrovascular burden in midlife predicts depressive symptomatology in later life [21], underscoring a strong association between cerebrovascular burden and major depressive disorder (MDD) [5]. Additional evidence for the cerebrovascular accident (CVA)-related onset of MDD derives from findings indicating that elderly individuals frequently show no history of depressive episodes prior to experiencing CVA events [21,22,23].

Cerebral small vessel disease (CSVD) also plays a pivotal role in the context of CVA. CSVD encompasses a spectrum of conditions resulting from damage to small blood vessels in the brain. It is typically characterized by white matter hyperintensities, lacunes of presumed vascular origin, cerebral microbleeds, enlarged perivascular spaces, and global cerebral atrophy. Closely linked to aging and vascular risk factors, CSVD has been identified as a major contributor to morbidity in ischemic and hemorrhagic stroke, dementia, and depression [24].

Furthermore, late-life depression is associated with several comorbidities that contribute to its pathological burden, including CSVD and beta-amyloid deposition, both of which have been associated with alterations in brain networks and cortical thinning [25]. Studies using magnetic resonance imaging (MRI) have suggested the term vascular depression focusing on the presence and severity of changes in white matter or subcortical gray matter lesions. These studies support the hypothesis that depressive symptoms are associated with brain volume loss, decreased white matter integrity, and lesions in fronto-striatal-limbic regions [23,26,27]. However, not all post-CVA depression exhibits evident vascular burden, some individuals develop depressive symptoms despite minimal white matter changes, suggesting that vascular pathology alone is neither necessary nor sufficient for post-CVA depression, and that multiple pathogenic pathways exist [28]. Large-scale lesion-symptom mapping studies demonstrate distinct depressive symptom domains associated with specific lesion locations, while population-based meta-analyses report that approximately one-third of stroke survivors develop depression while two-thirds do not, demonstrating substantial heterogeneity in post-stroke depression outcomes [29]. Depression-related pro-inflammatory reactions are closely associated with endothelial dysfunction and promote arteriosclerosis, thereby increasing the risk of developing CSVD in major depression, however their relationship to post-CVA is conditional rather than deterministic [28]. Several cardiovascular risk factors such as hyperglycemia, hypertension, dyslipidemia, smoking, homocysteinemia, and diabetes may contribute to vascular depression [23,30]. In clinical practice, vascular depression is tentatively diagnosed when depression emerges temporally approximate to documented cerebrovascular events or neuroimaging evidence of white matter disease, though no consensus diagnostic criteria exist and diagnosis is often retrospective [28]. Inflammation is also recognized as a canonical response to hypoxia and is therefore closely tied to neovascularization. While inflammation plays essential protective and regenerative roles, excessive or dysregulated inflammatory activity can drive the onset and progression of acute and chronic disorders that impair cerebrovascular function [31]. A substantial number of randomized controlled trials have investigated the effects of anti-inflammatory treatments on depression. Notably, these studies have demonstrated the efficacy of anti-inflammatory agents in alleviating depressive symptoms [24].

## 5. Microglial Activation, CVA and MDD

### 5.1. Microglial Activation Following CVA

Neuroinflammation is defined as an inflammatory response within the brain, mediated by cytokines, chemokines, reactive oxygen species, and secondary messengers produced by microglia, astrocytes, and endothelial cells [32,33]. The blood–brain barrier (BBB) composed of endothelial cells connected by tight junctions (TJs), maintains selective permeability and brain homeostasis, and becomes comprised following stroke due to microglial activation, facilitating white blood cells into the CNS [34,35,36,37,38].

Microglia, also referred to as “the innate immune cells of the brain” or “resident macrophages of the central nervous system”, constitute essential components of the central nervous system, performing immune and physiological functions that are crucial for brain development, immune response, and maintenance of brain homeostasis. Throughout their lifespan, microglia express a diverse range of surface receptors, known as “sensome” [39]. These receptors allow them to detect various local and external stimuli, including soluble ligands, cells, extracellular matrix components, damage-associated molecular patterns (DAMPs), and pathogen-associated molecular patterns (PAMPs). Initially, microglia were identified based on their morphological profiles as “resting” and “activated”. These terms were used to describe cells morphologically under physiological (“resting”) versus pathological (“activated”) conditions [40,41].

Subsequently, microglia are classified according to functional profile as either M1 (pro-inflammatory) or M2 (anti-inflammatory) [42]. However, their complexity cannot be reduced to simplistic and polarized categories like “pro-inflammatory” versus “anti-inflammatory” or “M1” versus “M2”. Instead, microglia are highly dynamic and adaptable, remaining active according to life stage, CNS region, species, sex, and health status. This adaptability allows them to develop their functions in accordance with the specific cellular context. Microglia can alter their morphology, proteomic, transcriptomic, and metabolic profiles in response to various stimuli, both in healthy and diseased conditions [40]. This promotes different microglial states, including disease associated microglia (DMA), which are identified through single-cell RNA sequencing that reveals a specific transcriptional signature. Another type, referred to as dark microglia, is characterized by transmission electron microscopy (TEM), which shows an acidophilic cytoplasm [40,43]. scRNA-seq studies revealed diverse microglial transcriptional states associated with different physiological and pathological conditions, including DAM, major histocompatibility complex (MHC), interferon (IFN), and proliferative microglia states [44,45].

**Figure 1:**
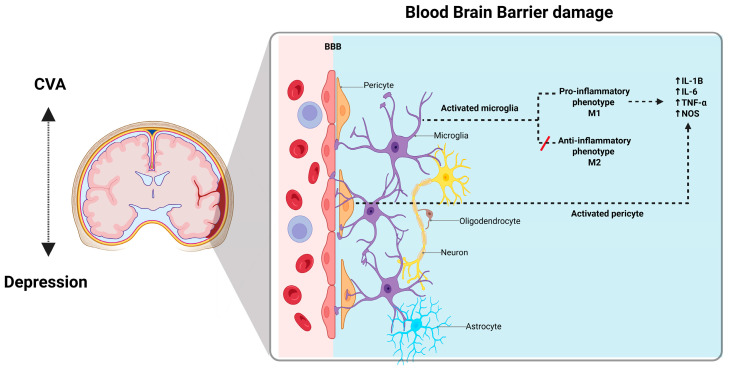
The blood-brain-barrier. BBB is composed of a continuous layer of endothelial cells connected by tight junctions (TJs), which help to form a selective physical barrier and maintain brain homeostasis. BBB disruption may also be strongly associated with the activation state of microglia. The microglia are classified according to their function in M1 (pro-inflammatory), or M2 (anti-inflammatory), and their integrity is greatly diminished by inflammatory microglia after a stroke. Neuroinflammatory response involves both infiltration of white blood cells into the central nervous system and the activation of resident microglia; this response in the central nervous system leads to injury. In infectious diseases and CVA the microglia release proinflammatory mediators that contribute to neuronal dysfunction and cell death. Created with BioRender.

When CVA occurs, inflammatory M1 microglia become activated and release pro-inflammatory mediators that contribute to neuronal dysfunction and cell death [38,46,47]. Endothelial cells and pericytes also play a significant role in sensing and propagating these inflammatory signals within the CNS [32]. Pericytes constitute a vital component of the BBB and have extensive contacts with endothelial cells that line the capillaries, astrocyte end feet that enclose cerebral vessels, perivascular microglia/macrophages, and parenchymal neurons [48,49]. They play a role in the immune response, including the disruption of the BBB induced by inflammation, the propagation of peripheral and central inflammation, and the expression of receptors that enable appropriate responses during inflammation-stimulated states [50,51]. It seems reasonable to posit that these immune properties of pericytes are important in their communication within the neurovascular unit, and that they provide a mechanism by which they participate in neuroinflammatory processes in brain infections and diseases [52]. (See Figure 1).

Both ischemic and hemorrhagic strokes lead to neuronal cell death, which in turn triggers the release of damage-associated molecular patterns (DAMPs) from dying cells, thereby inducing sterile inflammation. This localized brain inflammation also worsens the brain injury by promoting the blood–brain barrier disruption and microvascular dysfunction, which in turn exacerbate cerebral edema and oxidative stress. Consequently, DAMPs activate local immune cells, such as microglia, through pattern recognition receptors (PRRs), including toll-like receptors (TLRs) [53,54,55]. TLRs constitute an innate immune receptor family that plays a pivotal role in the neuroinflammatory process induced by stroke. A number of TLRs, including TLR2, TLR3, TLR4, TLR7, and TLR9, have been identified as playing a role in stroke outcomes [56,57]. It has been demonstrated that the expression of TLR2, TLR4, and TLR9 is increased following cerebral ischemic injury [46,54]. Moreover, TLR2 deficiency in mice has been shown to mitigate ischemic stroke–related symptoms [58,59].

**Figure 2:**
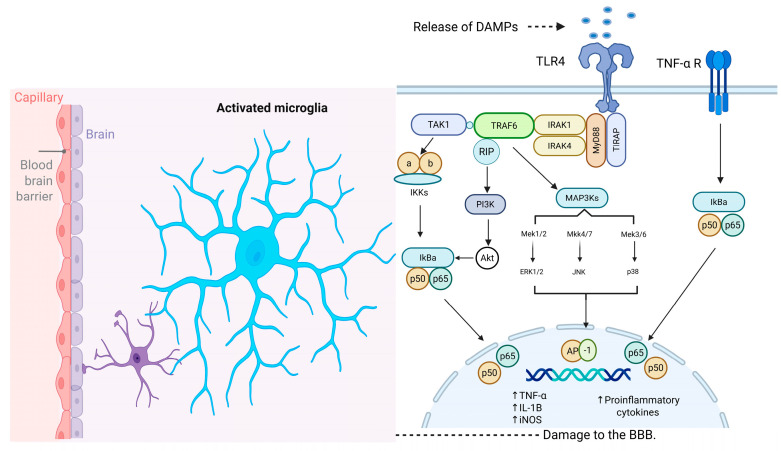
Molecular Mechanism Underlying Microglial Activation Following CVA. TLRs play critical roles in the activation of microglia during the neuroinflammatory response induced by damage-associated molecular patterns (DAMPs) in stroke. MyD88 recruits IRAK family kinases through its death domain (DD). IRAK proteins interact with TRAF-6, allowing TRAF-6 to activate the MAPKKK TAK1. TAK1 phosphorylates the IKK complex, activating it. The activated IKK complex phosphorylates IκB, marking it for degradation and thereby releasing NF-κB (p50/p65). Once released, it translocates into the nucleus, where it promotes the transcription of specific target genes. Additionally, TAK1 can stimulate the AP-1 pathway by activating the pathway of MAP kinases. TNF-α receptor activation induces the canonical pro-inflammatory transcriptional factors such as NF-κB and subsequent production of inflammatory mediators. Abbreviations: BBB, blood–brain barrier; DAMPs, damage-associated molecular patterns; TLR4, toll-like receptor 4; TNF-α R, tumor necrosis factor-alpha receptor; TAK1, transforming growth factor-beta-activated kinase 1; MyD88, adapter protein myeloid differentiation factor 88; IRAK, interleukin receptor associated kinase; TRAF6, tumor necrosis factor receptor-associated factor 6; TIRAP, toll-interleukin 1 receptor domain containing adaptor protein; MAP3K, mitogen-activated protein kinase kinase kinase; IKKs, IκB kinases; PI3K, phosphoinositide 3-kinase; IkBa, inhibitor of kappa light chain gene enhancer in B cells, alpha; Akt, v-akt murine thymoma viral oncogene homolog; Mek1/2, mitogen-activated protein kinase kinases 1/2; Mkk4/7, mitogen-activated protein kinase kinase 4/7; Mek3/6, mitogen-activated protein kinase kinases 3/6; ERK1/2, extracellular regulated kinase 1/2; JNK, c-Jun NH2-terminal kinase; AP-1, activator protein 1; TNF-α, tumor necrosis factor alpha; IL-1B, interleukin-10; iNOS, inducible nitric oxide synthase. Created with BioRender.

Studies on TLRs indicate that their activation leads to the recruitment of intracellular adaptor proteins—including myeloid differentiation primary response 88 (MyD88), TIR domain–containing adaptor-inducing interferon-β (TRIF), TIR domain–containing adaptor protein (TIRAP), and TRIF-related adaptor molecule (TRAM)—followed by the production of inflammatory mediators and type I interferons (IFNs). This occurs via MyD88- and/or TRIF-dependent pathways [60]. The pathway that requires MyD88-independent activation of nuclear factor κB (NF-κB) induces the production of inflammatory cytokines, including IL-1, IL-8 and TNF-α [56]. MyD88 possesses a TIR domain that is capable of binding to the TIR domain present on an activated receptor. Following its binding to the TLR, MyD88 recruits IRAK family kinases through its death domain (DD). Interleukin receptor-associated kinase (IRAK) interacts with tumor necrosis factor receptor-associated factor 6 (TRAF-6), thereby enabling TRAF-6 to activate the mitogen-activated protein kinase kinase kinase (MAP3K) TAK1. TAK1 phosphorylates the IKK complex, thereby activating it. The activated IKK complex phosphorylates IκB, the inhibitor of NF-κB, targeting it for proteasomal degradation. This degradation releases NF-κB, allowing it to translocate into the nucleus and promote the transcription of specific target genes. Furthermore, TAK1 can stimulate the AP-1 pathway by activating the pathway of MAP kinases [60,61,62] (See Figure 2).

Accordingly, an augmentation of Toll-like receptor 4 (TLR4) on microglia initiates a signal transduction cascade that modulates nuclear factor kappa-light-chain-enhancer of activated B cells (NF-κB)-mediated proinflammatory gene expression, which may ultimately culminate in neuronal cell death [36,38]. It is noteworthy that NF-κB plays a key role in regulating numerous proinflammatory cytokines, including TNF-α and IL-1β, in a multitude of pathological conditions, such as CVA [56]. The IL-1β cytokine serves to amplify the inflammatory signal by activating other cells that possess the corresponding receptors, which are crucial signals for the recruitment of leukocytes to the injury site [57]. Moreover, the activation of the MAC1 receptor also results in phagocytic activity and the activation of NADPH oxidase and the production of ROS, as well as the release of TNF-α [63,64]. TNF-α serves a central role in initiating and regulating the inflammatory cascade and is produced predominantly by activated microglia in response to TLR activation [62,65,66].

Although there is no direct evidence of the relationship between MyD88 and CVA-MDD, evidence from bioinformatic analyses demonstrates the upregulation of MYD88 along with STAT1 and CASP8 genes in ischemic stroke, highlighting the properties of these genes as potential therapeutic targets [67]. Remarkably, targeting MyD88 ameliorates the injury caused by ischemic insults [68]. Furthermore, an enriched environment promoted a neuroprotective effect after traumatic brain injury by downregulating NF-kB signaling [69]. Since MyD88 contributes to the activation of this pathway, this information suggests that MyD88 inhibition could represent a potential therapeutic strategy to treat brain injury derived from CVA or mechanical insults by downregulating the TLR-NFKB axis. Ultimately, the inhibition of MyD88 could contribute to the amelioration of depression derived from these types of brain damage given that upstream elements of MyD88 such as TLRs are also upregulated in postmortem samples of patients with depression [70].

Promising findings demonstrate that MyD88 inhibitor TJ-M2010-2 ablates the depressive-like behaviors and confers protection against ischemic insults in rodents [71,72], highlighting its potential as a key therapeutic target for treating cerebrovascular accidents and the associated depression. Notably, systemic delivery of TJ-M2010-2 does not compromise BBB integrity and inhibits microglial activation [72], offering a crucial advantage that future drugs will need to incorporate to represent a viable therapeutic strategy.

### 5.2. The Inflammatory Response and MDD

MDD has been associated with an inflammatory response mediated by pro-inflammatory cytokines [73,74], including interleukins IL-12 and IL-6 [73,75,76,77,78], TNF-α [78,79,80], and acute phase reactants such as C-reactive protein and haptoglobin [80,81]. Some studies [79,80,82], have documented significantly higher concentrations of cytokines IL-6 and TNF-α in serum and plasma, in patients with MDD compared to healthy controls, supporting the hypothesis that these cytokines play a crucial role in the pathogenesis of MDD. The diverse effects of the inflammatory response are elucidated through the distinct recruitment of signaling complexes after ligand binding. TNF-α receptor activation induces NF-κB dependent transcription of proinflammatory mediators, which may contribute to behavioral effects associated with MDD [53,62,68,69,83,84]. Furthermore, TNF-α and IL-6 have been linked to chronicity and severity of MDD [85,86,87]. Collectively, these findings underscore the significant role of inflammation in the MDD pathophysiology; however, current evidence does not clarify whether the activation of cytokines is a cause or a consequence of depressive episodes. Furthermore, inflammation is unlikely to be a necessary or sufficient mechanism on its own.

### 5.3. Microglial Activation and MDD

As previously mentioned, both ischemic and hemorrhagic strokes can lead to neuronal cell death, subsequently promoting the release of DAMPs and initiating microglial activation (Figure 2). The consequence of these events is neuroinflammation, which in turn trigger other negative effects such as blood–brain barrier disruption, microvascular dysfunction, exacerbated edema and oxidative stress. Thus, neuroinflammation constitutes a critical link between CVA–induced microglial activation and MDD.

Microglial activation is recognized as a central driver in the pathophysiology of MDD, often leading to the conceptualization of MDD as a microglial disease [88]. Particularly, the chronic activation and polarization towards the proinflammatory M1 phenotype result in chronic neuroinflammation and subsequent injury [89]. These activated microglia release proinflammatory cytokines, such as IL-1, IL-6, and TNF-α, which induce neuronal toxicity and reduce neurogenesis [89]. Moreover, microglial dysfunction compromises essential neuroplasticity mechanisms, including aberrant synaptic pruning and the breakdown of neuronal network integrity, a phenomenon likely mediated by excessive M1 activation and diminished M2 (anti-inflammatory) function [88]. Within this framework, modulating inflammatory pathways to reprogram the microglial phenotype has been advanced as a novel translational strategy for treating MDD through the targeted regulation of neuroinflammation [62]. Indeed, several antidepressants have been evaluated in stress-induced depression models to demonstrate their efficacy in restoring microglial function [90].

**Figure 3:**
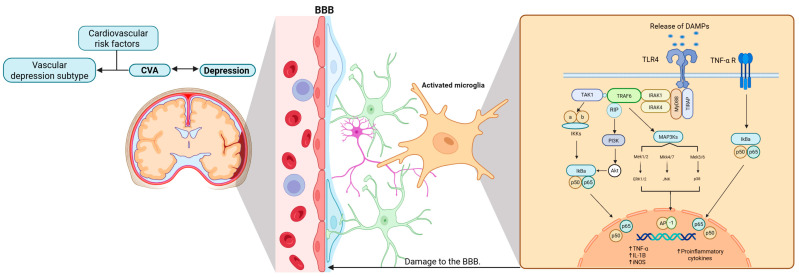
Microglial activation in cerebrovascular accidents and the manifestation of MDD. This figure illustrates the close relationship between CVA, microglial activation, and inflammation, and how these processes may potentially underlie the pathophysiology of depression. In particular, the vascular depression subtype is characterized by the presence of cardiovascular risk factors. Abbreviations: BBB, blood–brain barrier; DAMPs, damage-associated molecular patterns; TLR4, toll-like receptor 4; TNF-α R, tumor necrosis factor-alpha receptor; TAK1, transforming growth factor-beta-activated kinase 1; MyD88, adapter protein myeloid differentiation factor 88; IRAK, interleukin receptor associated kinase; TRAF6, tumor necrosis factor receptor-associated factor 6; TIRAP, toll-interleukin 1 receptor domain containing adaptor protein; MAP3K, mitogen-activated protein kinase kinase kinase; IKKs, IκB kinases; PI3K, phosphoinositide 3-kinase; IkBa, inhibitor of kappa light chain gene enhancer in B cells, alpha; Akt, v-akt murine thymoma viral oncogene homolog; Mek1/2, mitogen-activated protein kinase kinases 1/2; Mkk4/7, mitogen-activated protein kinase kinase 4/7; Mek3/6, mitogen-activated protein kinase kinases 3/6; ERK1/2, extracellular regulated kinase 1/2; JNK, c-Jun NH2-terminal kinase; AP-1, activator protein 1; TNF-α, tumor necrosis factor alpha; IL-1B, interleukin-10; iNOS, inducible nitric oxide synthase. Created with BioRender.

The molecular mechanisms underlying microglial activation are crucial for understanding the relationship between CVA and MDD, which in turn supports the vascular depression hypothesis. Furthermore, the current literature emphasizes the fundamental role of the immune system in this intricate and complex pathogenic process. These elements are collectively summarized in Figure 3.

### 5.4. Depression as a Risk Factor for Stroke

We have reviewed the pathophysiology underlying post-stroke depression; however, it is worth briefly mentioning the inverse scenario, in which depression is considered a risk factor for a cerebrovascular event. Several meta-analyses have demonstrated that depression is associated with a significantly increased risk of stroke. Specifically, a positive association has been identified for both fatal and ischemic events [91]. Notably, the magnitude of this association is comparable to that observed for traditional risk factors such as smoking and obesity [92].

Moreover, this elevated risk appears to be independent of sex and conventional cardiovascular risk factors, including hypertension and diabetes mellitus [93]. Longitudinal data also suggest a prodromal phase: individuals who later experienced a stroke showed a worsening of depressive symptoms up to 12 months before the ischemic event [94]. Conversely, patients who develop post-stroke depression face a higher risk of recurrence and exhibit significant declines across multiple cognitive domains [95].

## 6. Conclusions

The evidence demonstrates a clear association between CVA and MDD, characterized by elevated levels of proinflammatory cytokines, a dysregulated immune response, and heightened microglial activation. In light of current literature, it is imperative that future studies expand knowledge of key elements such as MyD88, which is critically involved in the dysregulation of microglial homeostasis and, consequently, in the pathogenesis of both CVA and MDD.

Ultimately, this review highlights cerebrovascular pathology as a critical risk factor for the development of MDD. The predictive value of midlife cerebrovascular burden for later depressive symptoms, together with evidence that MDD frequently emerges de novo following CVA in older adults, underscores the vascular contribution to depressive pathogenesis and provides strong support for the vascular depression hypothesis.

### Limitations

This review was conceived as a narrative, non-systematic synthesis; therefore, we did not conduct a PRISMA-style study selection flow or quantify the number of records retained in each subsection. Instead, for each thematic area we used iterative, citation-chaining searches in PubMed and Scopus (English and Spanish, no date limits) to prioritize recent high-quality primary studies and landmark reviews most relevant to the mechanistic questions of interest. We did not formally search gray literature or unpublished studies, and we now acknowledge this as a limitation of the work.

## Data Availability

No new data were created or analyzed in this study. Data sharing is not applicable to this article.

## Abbreviations

AP-1	Activator Protein 1
ASC-1	Apoptosis-associated speck-like protein containing a C-terminal caspase recruitment domain
BBB	Blood–Brain Barrier
CNS	Central Nervous System
COX-2	Cyclooxygenase-2
CRP	C-reactive protein
CSVD	Cerebral Small Vessel Disease
CVA	Cerebrovascular Accident
DALYs	Disability-Adjusted Life Years
IFNs	Type I Interferons
IKK	IκB kinase complex
IL-1, IL-6, IL-8, IL-12, IL-18	Interleukin 1, 6, 8, 12, 18
iNOS	Inducible Nitric Oxide Synthase
IRAK	Interleukin receptor-associated kinase
IκB	Inhibitor of NF-κB
LPS	Lipopolysaccharides
MAC1	Macrophage-1 antigen or complement receptor 1 (CD11b/CD18)
MAP3K/TAK1	Mitogen-Activated Protein Kinase Kinase Kinase
MDD	Major Depressive Disorder
MHC-II	Major Histocompatibility Complex II
MRI	Magnetic Resonance Imaging
MyD88	Myeloid Differentiation Factor 88
NF-κB	Nuclear Factor κB
NLR	Nod-like receptor
NLRP3	Nod-like receptor family pyrin domain containing 3
TIRAP	TIR domain-containing adaptor protein
TJs	Tight Junctions
TLR	Toll-like Receptor
TNF-α	Tumor Necrosis Factor alpha
TRAF-6	Tumor necrosis factor receptor-associated factor 6
TRAM	TRIF-related adaptor molecule
TRIF	TIR domain-containing adaptor-inducing interferons
